# Effects of artificial defoliation on growth and biomass accumulation in short-rotation sweetgum *(Liquidambar styraciflua)* in North Carolina

**DOI:** 10.1093/jis/14.1.107

**Published:** 2014-08-12

**Authors:** Robert M. Jetton, Daniel J. Robison

**Affiliations:** 1 Camcore, Department of Forestry and Environmental Resources, North Carolina State University, Campus Box 8008, Raleigh, NC 27695-8008; 2 Davis College of Agriculture, Natural Resources, and Design, West Virginia University, P.O. Box 6108, Morgantown, WV 26506-6108. Formerly, College of Natural Resources and Department of Forestry and Environmental Resources, North Carolina State University, Campus Box 8001, Raleigh, NC 27695-8001

**Keywords:** simulated herbivory, forest plantation, short-rotation woody crop

## Abstract

Sweetgum, *Liquidambar styraciflua* L. (Hamamelidales: Hamamelidaceae), is a species of interest for short-rotation plantation forestry in the southeastern United States. Despite its high levels of resistance to many native insects and pathogens, the species is susceptible to generalist defoliators during outbreak epidemics. The objective of this field study was to evaluate the potential impact of defoliation on sweetgum growth and productivity within the context of an operational plantation. Over three growing seasons, trees were subjected to artificial defoliation treatments of various intensity (control = 0% defoliation; low intensity = 33% defoliation; moderate intensity = 67% defoliation; high intensity = 99% defoliation) and frequency (not defoliated; defoliated once in April of the first growing season; defoliated twice, once in April of the first growing season and again in April of the second growing season). The responses of stem height, stem diameter, stem volume, crown volume, total biomass accumulation, and branch growth were measured in November of each growing season. At the end of the first growing season, when trees had received single defoliations, significant reductions in all growth traits followed the most severe (99%) defoliation treatment only. After the second and third growing seasons, when trees had received one or two defoliations of varying intensity, stem diameter and volume and total tree biomass were reduced significantly by 67 and 99% defoliation, while reductions in stem height and crown volume followed the 99% treatment only. All growth traits other than crown volume were reduced significantly by two defoliations but not one defoliation. Results indicate that sweetgum is highly resilient to single defoliations of low, moderate, and high intensity. However, during the three-year period of the study, repeated high-intensity defoliation caused significant reductions in growth and productivity that could have lasting impacts on yield throughout a harvest rotation.

## Introduction

Intensively managed short-rotation hardwood plantations in the southern United States offer opportunities to meet a diversity of management objectives that cannot be met through natural stand management alone ( Robison et al. 1998 ; Romagosa and Robison 2003 ). Initial interest in hardwood plantation forestry coincided with increasing demand for high quality and easily accessible sources of hardwood fiber for the production of paper products ( Kellison 1997 ; Steinbeck 1999 ). More recently, management objectives have been expanded to include biomass for energy production, carbon mitigation, and environmental enhancement on former agricultural lands ( Graham et al. 1992 ; Tolbert and Schiller 1995 ; Volk et al. 2004 ). A number of hardwood species have been evaluated for use in such silvicultural systems in the region with sweetgum ( *Liquidambar styraciflua* L.), cottonwood ( *Populus deltoides* Bartr. ex Marsh.), sycamore ( *Platanus occidentalis* L.), and more recently *Eucalyptus* spp. having emerged as the most promising ( Kellison 1997 ; Steinbeck 1999 ; Gonzalez et al. 2010 ).

Sweetgum was recognized early on as a prime candidate for short-rotation applications in the southern U.S. due to its wide site adaptability, potential for genetic improvement, responsiveness to intensive silvicultural inputs, and high level of native pest resistance ( Kormanik 1990 ; HRC 1997; Steinbeck 1999 ). During the ensuing decades, the species has continued to receive considerable interest as a short-rotation species with respect to tree improvement ( Birks 2001 ; HRC 2001), intensive silviculture ( Guo et al. 1998 ; Samuelson et al. 2001 ), vegetative propagation ( Gocke 2006 ), and biomass production and carbon sequestration ( Davis and Trettin 2006 ; Nyakatawa et al. 2006 ; Williams and Gresham 2006 ; Sanchezet al. 2007 ; Cobb et al. 2008 ; Coyle et al. 2008 ). Currently, there are approximately 8,000 ha of sweetgum plantations in cultivation across the southern U.S. established as sawtimber, pulpwood, and bioenergy crops ( Wright and Cunningham 2008 ).

Insect herbivory can have a number of negative effects on natural and planted forests, including reduced growth and survival, increased susceptibility to secondary insect pests and disease, and rotation delays (Kulman 1971). Compared with a variety of other hardwood species, sweetgum has relatively few problems with insect pests and disease ( Ward and Sayer 2004 ). However, despite its relatively high level of native pest resistance, sweetgum remains susceptible to defoliation by generalist folivores, such as the gypsy moth ( *Lymantria dispar* L.) and the forest tent caterpillar ( *Malacosoma disstria* Hübner). These forest pests typically exist at low population density but have the capacity to enter eruptive outbreak cycles over wide areas with high population density and severe defoliation intensity that can last for several years ( Drooz 1985 ). Sweetgum is highly preferred by gypsy moth and is an important component of natural stands most susceptible to defoliation by this pest throughout the eastern U.S. ( Barbosa et al. 1983 ; Davidson et al. 2001 ). Sweetgum is also a common host of the forest tent caterpillar throughout the southeastern U.S. ( Kormanik 1990 ) and has been defoliated completely in natural stands during outbreaks ( Leininger and Solomon 1995 ). Given the investment in current plantings, the gypsy moth’s continued spread into the southeastern U.S., and the ubiquitous nature of the forest tent caterpillar in the region, the risk for widespread and prolonged infestation and loss in sweetgum plantations is real.

In short-rotation systems that utilize *Populus* spp. and hybrids, much useful research has been conducted on the major defoliating insects of concern. These include the cottonwood leaf beetle *(Chrysomela scripta* F), the forest tent caterpillar, and the gypsy moth. Studies have examined the effects of real and simulated herbivory on tree growth and biomass accumulation ( Bassman et al. 1982 ; Solomon 1985 ; Reichenbacker et al. 1996 ; Kosola et al. 2001 ; Coyle et al. 2002 ), the preference and performance of larval and adult insects among hybrids and clones ( Caldbeck et al. 1978 ; Bingaman and Hart 1992 ; Robison and Raffa 1994 , 1997; Coyle et al. 2001 , 2003), and the response of larval insects to transgenic cultivars ( Robison et al. 1994 ).

Relative to *Populus* spp., very little information is available on the responses of sweetgum to real or simulated insect defoliation within operational forest plantations managed for fiber and biomass. Most studies have focused on the impact of defoliation in mixed-species natural stands ( Leininger and Solomon 1995 ; Davidson et al. 2001 ; Eisenbies et al. 2007) or in young ( **<** one-year-old) containerized seedlings in the nursery or greenhouse setting ( Chambers and Smith 1982 ; Birks 2001 ; Ward and Sayer 2004 ). Results show that sweetgum growth and crown condition recover quickly following outbreak collapse ( Leininger and Solomon 1995 ) and that the species tends to be at lower risk of mortality due to defoliation compared with more susceptible *Quercus* species ( Davidson et al. 2001 ; Eisenbies et al. 2007 ). Meanwhile, studies on young containerized seedlings show that real or simulated insect herbivory alone ( Chambers and Smith 1982 ) or in combination with other environmental stresses ( Birks 2001 ; Ward and Sayer 2004 ) can impact significantly all growth traits of weetgum, with stem height being the least affected ( Chambers and Smith 1982 ; Birks 2001 ). It also has been demonstrated in laboratory tests with foliage from progeny trials and greenhouse seedlings that the preference and performance of forest tent caterpillars varies among open-pollinated sweetgum families ( Jetton 2002 ).

While the field and greenhouse studies cited above are informative, they offer little evidence as to how sweetgum might respond to defoliation in an intensively managed plantation. Therefore, the objective of the current study was to determine the response of sweetgum growth and biomass accumulation to artificial defoliation within the context of an intensively managed short-rotation plantation in the coastal plain of North Carolina. The growth responses of trees subjected to defoliation treatments of varying intensity and frequency were measured over three growing seasons.

## Materials and Methods

### Study site

This study was conducted over three growing seasons in a 200 ha operational sweetgum plantation (International Paper Co., www.internationalpaper.com ) on an upland, coastal plain site in Hertford County, North Carolina (36.348370N and -76.840990W). Soils were moderately well-drained with fine loamy sand surface over clay subsoil typical of the Craven series (USDA 1984). The previous mixed pine-hardwood stand was clearcut, and after site preparation consisting of bedding and chemical competition control, the sweetgum plantation was established with 1-0 seedlings planted at 3.0 by 2.5 m spacing. Seedlings were from an International Paper Co. seed orchard mix containing genotypes having superior growth performance in progeny tests. The plantation received annual fertilization (diammonium-phosphate at 224 kg/ha and urea at 224 kg/ha) and herbicide (unknown rates sulfometuron methyl [Oust!]/glyphosate [Accord!] mixture) treatments. The artificial defoliation study was superimposed on the plantation when the trees were beginning their second growing season in the field. The study plot was located centrally within the plantation with an approximate 200 m buffer from nearby roads.

### Study design and treatments

The experiment was a randomized complete block with four blocks (12 trees per block), four artificial defoliation intensity treatments including the control, and three trees per treatment per block for a total of 48 trees. The defoliation intensity treatments were applied first in April of Year 1 and consisted of removing 0 (control), 33 (low intensity), 67 (moderate intensity), or 99% (high intensity) of each leaf with scissors. For each treatment, the new outer crown leaves (fully unfolded and expanded) were clipped first, followed by the inner crown leaves, then by the newly emerged leaves (not fully unfolded or expanded) and buds at the crown margins. This three-part process on each tree was conducted over a two-week period and was intended to mimic the spatial and temporal pattern of progressive feeding by gypsy moth or forest tent caterpillar populations.

In April of Year 2, the blocks were split and half of the trees that received defoliation treatments in April of Year 1 were selected randomly to receive a second artificial defoliation, each at the same intensity as applied the previous year. This resulted in seven classes of defoliation frequency and intensity with 12 trees for the control and six trees for each defoliation class, namely trees that received one 33, 67, or 99% defoliation (in April ofYear 1) and trees that received two 33, 67, or 99% defoliations (once in April of Year 1 and again in April of Year 2).

### Measurements and statistical analysis

Study trees were measured in April of Year 1 (pre-treatment) before the artificial defoliation treatments and again post-treatment at the end of each growing season of the study (i.e., November of Years 1, 2, and 3). Growth traits measured were stem height (m) and stem diameter (cm) at 15 cm above ground level. Calculated growth parameters reported include crown volume (m ^3^ ), stem volume (m ^3^ ), and total biomass (kg). Crown volume was estimated from the volume equation for a cone (= π*crown basal radius ^2^ *0.33*crown height). Stem volume (= (0.0000339*stem diame-ter ^2^ *stem height) + 0.00263) and total biomass (= (0.0305*stem diameter ^2^ *stem height) + 3.788) were calculated with equations developed for sweetgum by Williams and Gresham (2006) . The length (cm) and diameter (mm) of five mid-crown branches on the south-facing side of each tree were measured at the end of Years 1 and 2.

Analysis of variance (ANOVA) was performed by using the PROC GLM procedure of SAS version 9.1 ( SAS institute 2003 ). After Year 1, when trees had received a single artificial defoliation treatment, the main effects of block and defoliation intensity (0, 33, 67, or 99%) and the interaction of block and defoliation intensity were assessed for the stem, crown, and biomass growth traits. After the end of Year 3, when the blocks had been split and trees had received either one or two artificial defoliation treatments, the main effects of block, defoliation intensity (0, 33, 67, or 99%), defoliation frequency (0, 1, or 2), and year (Year 2 or Year 3) and all two-way interactions were tested for the stem, crown, and biomass growth traits in the Year 2 and Year 3 combined data. Branch growth traits were analyzed with the Year 1 and Year 2 data. The Year 1 analysis assessed the main effects of block and defoliation intensity and the interaction of block and defoliation intensity for both branch length and diameter. The Year 2 analysis tested the main effects of block, defoliation intensity, defoliation frequency, and all two-way interactions. In all analyses, when significant differences among the defoliation intensities and frequencies were found, means were compared by using the Tukey-Kramer method at the α = 0.05 significance level ( SAS Institute 2003 ).

## Results

At the end of Year 1, when the trees had received a single artificial defoliation treatment of varying intensity the previous April, stem height was significantly affected by the main effects of block and defoliation intensity, while stem diameter, crown volume, stem volume, and total biomass were affected significantly by defoliation intensity alone ( Table 1 ). Among the varying defoliation intensities, stem height and crown volume decreased with increasing intensity, while stem diameter increased slightly from 0% to 33% defoliation and then decreased with increasing intensity ( Table 2 ). This same trend was reflected in the calculated parameters of stem volume and total biomass. For all growth traits, mean com-comparison analyses indicated that among the varying defoliation intensities, only those trees defoliated at the 99% level were significantly smaller than the 0% defoliation con-controls ( Table 2 ).

**Table 1.**
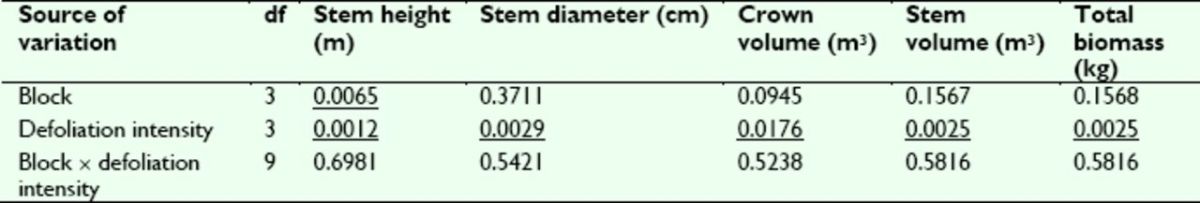
Analysis of variance *P* -values for sweetgum growth and biomass traits assessed following Year 1 of the artificial defoliation study.

*P* -values are reported for each main and interaction effect included in the ANOVA model; underlined *P* -values are significant at α ≤ 0.05.

**Table 2.**
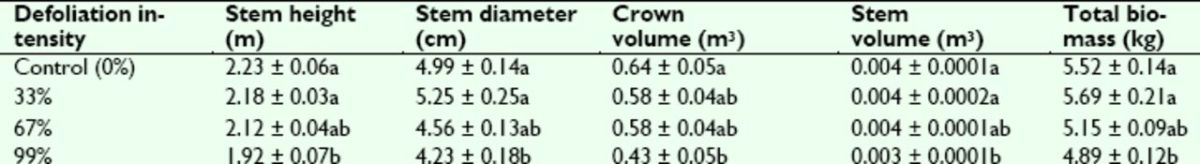
Means (± SE) for sweetgum growth and biomass traits following Year 1 of the artificial defoliation study.

Data reflect the responses of sweetgum growth one growing season after a single artificial defoliation treatment of varying intensity. Means within a column followed by different lowercase letters are significantly different at α ≤0.05 by *F* -statistic protected Tukey’s studentized range test.

At the end of Year 3, when the blocks had been split and trees had received either one (in April of Year 1) or two (in April of Years 1 and 2) artificial defoliation treatments of varying intensity, stem height, stem diameter, stem volume, and total biomass were affected significantly by the main effects of block, defoli-defoliation intensity, defoliation frequency, and year, while crown volume was affected significantly by block, defoliation intensity, and year, but not by defoliation frequency ( Table 3 ). Significant interactions between the effects of block and defoliation frequency were detected for stem height, stem volume, and total biomass.

**Table 3.**
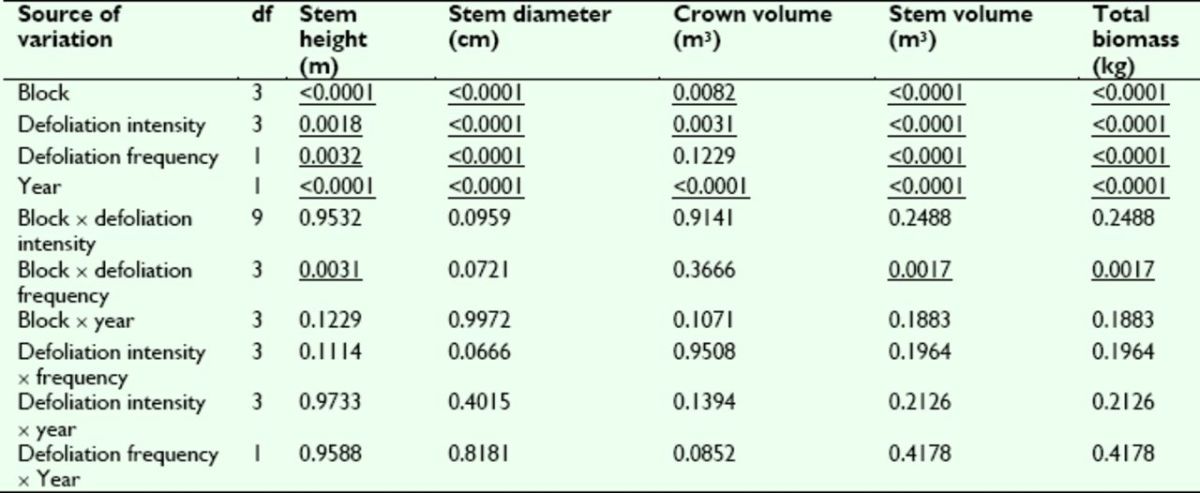
Analysis of variance *P* -values for sweetgum growth and biomass traits assessed following Year 3 of the artificial defoliation study.

*P* -values are reported for each main and interaction effect included in the ANOVA model. Underlined *P* -values are significant at α ≤ 0.05.

Similar to the end of Year 1, among the varying defoliation intensities, stem height and crown volume decreased with increasing intensity, while stem diameter, stem volume, and total biomass increased slightly from 0% to 33% defoliation and then decreased with increasing intensity ( Table 4 ). Mean comparison analysis indicated that stem height was significantly smaller at the 99% defoliation intensity relative to the 0% defoliation control and that stem diameter, crown volume, stem volume, and total biomass were significantly smaller relative to the control at both the 67% and 99% defoliation intensities.

**Table 4.**
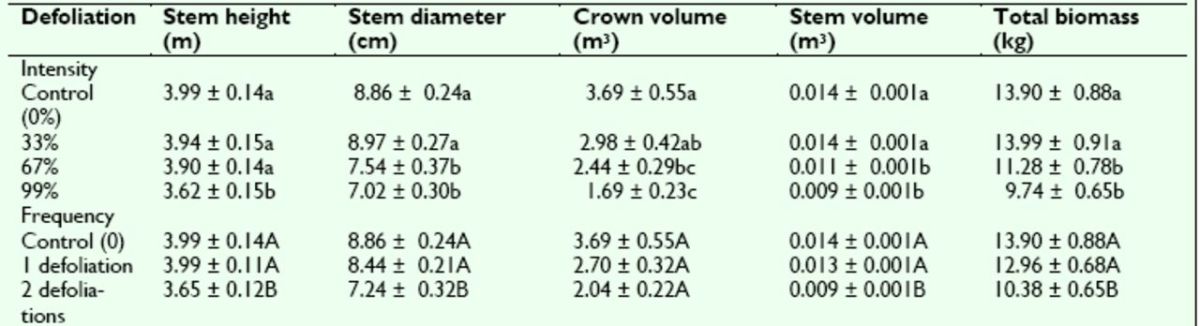
Means (± SE) for sweetgum growth and biomass traits following Years 2 and 3 of the artificial defoliation study.

Data reflect the responses of sweetgum growth over three growing seasons after artificial defoliation treatments of varying intensity and frequency. Means within a column followed by different lowercase letters (defoliation intensity) or uppercase letters (defoliation frequency) are significantly different at α ≤0.05 by *F* -st atistic protected Tukey’s studentized range test.

For defoliation frequency, stem height, stem diameter, stem volume, and total biomass decreased as the number of defoliation treatments increased. Mean comparison analysis indicated that trees receiving two defoliation treatments were significantly smaller than the controls for each trait, while those receiving one defoliation treatment did not differ from the control ( Table 4 ). Crown volume followed the same general trend but did not differ significantly with defoliation frequency.

**Fig 1.**
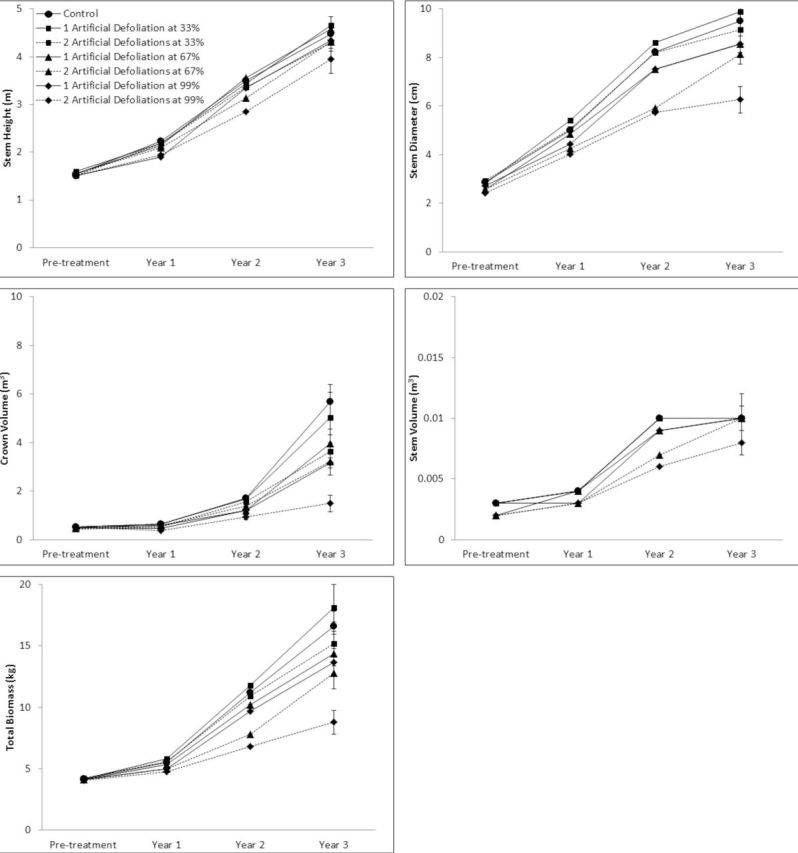
Response trajectories of sweetgum growth and biomass traits in each defoliation frequency/intensity class over the three-year duration of the artificial defoliation study. A high quality figure is available online.

On average, trees increased in size from Year 2 to Year 3 despite artificial defoliation treatments. Stem height increased from 3.33 (± 0.06) to 4.39 (± 0.08) m, stem diameter from 7.50 (± 0.23) to 8.69 (± 0.22) cm, crown volume from 1.42 (± 0.06) to 3.98 (± 0.32) m ^3^ , tem volume from 0.009 (± 0.0004) to 0.01 (± 0.0007) m ^3^ , and total biomass from 9.95 (± 0.38) to 14.51 (± 0.65) kg ( Fig. 1 ).

Branch growth responded to artificial defoliation in a similar fashion to the other growth traits at the end of Year 1 ( Tables 5 and 6 ). Following a single defoliation treatment the previous April, both branch length and diameter were affected significantly by defoliation intensity. They decreased slightly with increasing defoliation severity and showed statistically significant reductions in growth relative to the controls after the 99% defoliation. Branch growth trends were the same following Year 2 ( Tables 5 and 6 ), with significant effects of 99% defoliation intensity on length and diameter. However, similar to crown volume at the end of Year 3, branch rowth at the end of Year 2 was not affected by defoliation frequency.

**Table 5.**
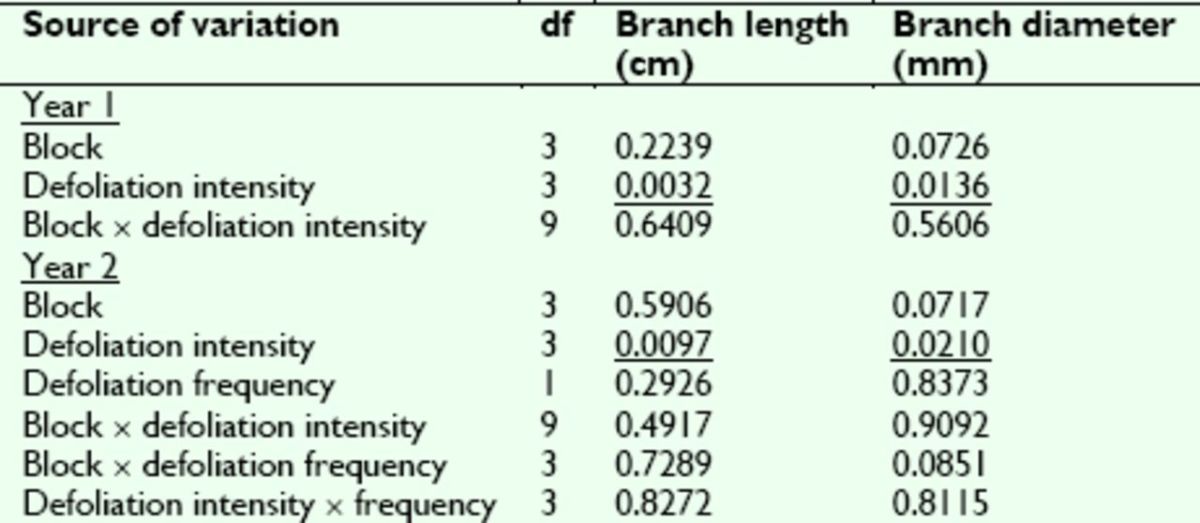
Analysis of variance *P* -values for sweetgum branch growth traits assessed following Year 1 and Year 2 of the artificial efoliation study.

*P* -values are reported for each main and interaction effect included in the ANOVA model. Underlined *P* -values are significant at α 0.05.

**Table 6.**
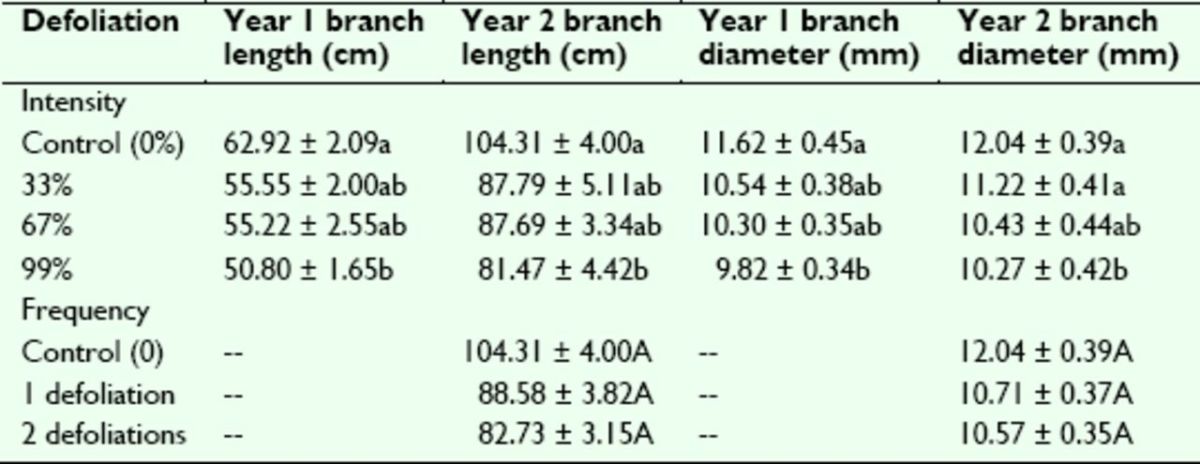
Means (± SE) for sweetgum branch growth traits following Years 1 and 2 of the artificial defoliation study.

Data reflect the responses of sweetgum growth over two growing seasons after artificial defoliation treatments of varying intensity and frequency. Means within a column followed by different lowercase letters (defoliation intensity) or uppercase letters (defoliation frequency) are significantly different at α ≤ 0.05 by *F* -statistic protected Tukey’s studentized range test.

## Discussion

It generally is accepted that many hardwood tree species have a high tolerance for one to a few years of insect defoliation at low to moderate intensity and that significant losses due to growth reduction, mortality, and rotation delays typically are associated with severe or repeated defoliations ( Kulman 1971 ). The results of this study indicate that, within the context of an intensively managed short-rotation plantation, the growth and productivity of artificially defoliated sweetgum responds to defoliation intensity and frequency as expected for a hardwood species. At the end of Year 1, significant reductions in all assessed growth and biomass traits were seen for the high (99%) defoliation intensity but not the low and moderate levels (33 and 67%, respectively). This same trend was seen for branch length and diameter at the end of Year 2 (the last year that branch growth was measured) and for stem height at the end of Year 3. The effects of defoliation intensity were more pronounced for stem diameter, crown volume, stem volume, and total biomass at the end of the third growing season when all four of these traits were reduced significantly by both moderate and high levels of artificial defoliation.

The response of sweetgum to artificial defoliation frequency indicates that, over the three-year duration of the field experiment, growth and biomass accumulation in plantation sweetgum were tolerant of a single artificial defoliation, regardless of intensity. At the end of Year 3, three full growing seasons after initial treatments and two growing seasons without foliage loss, there were no significant reductions in growth for any of the measured raits following a single defoliation event. It does not appear, however, that the growth and productivity of plantation sweetgum has the same level of tolerance following more frequent defoliation. The significant reductions in stem height, diameter, volume, and total biomass following two defoliation events suggest that one growing season free of defoliation is not enough for sweetgum to recover from the negative effects of multiple defoliations. Given that defoliators like the gypsy moth and the forest tent caterpillar often have outbreak phases that can last several years ( Drooz 1985 ), this does reflect a real-world scenario. Further, other sources of multiple-year defoliation, by insects, disease, high wind, or drought, may yield similar impacts on tree growth.

Artificial defoliation has been used to evaluate the growth and productivity responses of a number of tree species to foliage loss ( Coyle et al. 2005 ), and similar patterns of tolerance for low to moderate defoliation intensities/frequencies and increased susceptibility for moderate to high intensities/frequencies have been described for both hardwood *(Betula* spp., *Eucalyptus* spp., *Populus* spp.) and coniferous *(Abies* spp., *Larix* spp., *Pinus* spp.) species ( Bassman et al. 1982 ; Piene and Little 1990 ; Bassman and Zwier 1993 ; Krause and Raffa 1996 ; Reichenbacker et al. 1996 ; Kaitaniemi et al 1999; Anttonen et al. 2002 ; Pinkard et al. 2006 ). Many of these studies also report the general trend for decreased growth with increasing defoliation intensity, as seen for sweetgum in the current study ( Tables 2 and 4 ). Although this was the general trend, at the end of Years 1 and 3, stem diameter, stem volume, and total biomass all showed small increases in growth from the control (0%) to the low (33%) defoliation level before decreasing through the moderate and high intensity treatments. While it is temptingto suggest this result might indicate an overcompensation to low-intensity defoliation related to enhanced photosynthesis or within-tree resource redistribution, as described in other plant systems ( Trumble et al. 1993 ), these stem growth and biomass accumulation in-increases in response to 33% defoliation were not statistically significant and likely resulted from random variation, as suggested by Reichenbacker et al. (1996) , who found similar responses to low levels of artificial defoliation in clonal *Populus.*

The potential long-term effects of more frequent and severe defoliation on sweetgum growth and productivity are demonstrated most clearly by the growth trajectories of trees in each defoliation frequency/intensity class presented in Fig. 1 . For all five parameters, at the end of Year 3, it is clear that the growth of trees in the most severe defoliation frequency/intensity class (two artificial defoliation treatments at the 99% level) is lagging behind that of trees in the other classes. For stem height, this represents an 11% reduction in growth relative to the average height of trees in the other defoliation frequency/intensity classes. However, the height growth trajectory of trees receiving two 99% defoliation treatments appears to be parallel with the other classes, and this small difference is likely to become negligible over time, indicating that sweetgum height growth may have some level of tolerance for repeated severe defoliation. Other studies evaluating the effects of natural or simulated herbivory have reported that sweetgum height growth appears to be less susceptible to defoliation than other growth characteristics ( Chambers and Smith 1982 ; Birks 2001 ).

The potential long-term effects of repeated, high-intensity defoliation appear to be more pronounced for stem diameter, volume, and total biomass accumulation ( Fig. 1 ) than for other parameters. At the end of Year 3, growth and productivity reductions following two 99% defoliation treatments relative to averages of the other classes were 20 (stem volume), 30 (stem diameter), and 42% (total biomass). Unlike stem height growth, the growth trajectories for these parameters appear to be diverging from the other defoliation frequency/intensity classes, suggesting that repeated severe defoliator outbreaks may pose a serious threat to pulp and biomass yields in short-rotation sweetgum systems.

Overall, the results of this study suggest how intensively managed short-rotation sweetgum might respond to defoliation on a large scale. The data suggest that sweetgum can be expected to be tolerant of defoliation and insect control measures may be unnecessary in situations where monitoring indicates that increases in defoliator population density are expected to be of low intensity or a single occurrence. If survey data indicate a repeated high-density defoliator outbreak that could potentially result in severe defoliation intensity over multiple years, efforts to control insect populations through insecticides or other methods may be warranted and necessary to avoid substantial growth and productivity losses. However, because the data are derived from simulated defoliation, future research should focus on insect-mediated defoliation to more fully understand the interactions of sweetgum, insect defoliation, and short-rotation intensive silviculture.
